# SNP discovery and characterisation in White Rhino (*Ceratotherium simum*) with application to parentage assignment

**DOI:** 10.1590/1678-4685-GMB-2016-0058

**Published:** 2017-02-06

**Authors:** Christiaan Labuschagne, Desiré L. Dalton, J. Paul Grobler, Antoinette Kotzé

**Affiliations:** 1 Department of Genetics, University of the Free State, Bloemfontein, Free State, South Africa; 2 National Zoological Gardens of South Africa, Pretoria, Gauteng, South Africa; 3 Inqaba Biotechnical Industries (Pty) Ltd, Pretoria, Gauteng, South Africa

**Keywords:** Single nucleotide polymorphisms, microsatellite markers, White Rhinoceros, Ceratotherium simum

## Abstract

The white rhino is one of the great success stories of modern wildlife conservation, growing from as few as 50-100 animals in the 1880s, to approximately 20,000 white rhinoceros remaining today. However, illegal trade in conservational rhinoceros horns is adding constant pressure on remaining populations. Captive management of *ex situ* populations of endangered species using molecular methods can contribute to improving the management of the species. Here we compare for the first time the utility of 33 Single Nucleotide Polymorphisms (SNPs) and nine microsatellites (MS) in isolation and in combination for assigning parentage in captive White Rhinoceros. We found that a combined dataset of SNPs and microsatellites was most informative with the highest confidence level. This study thus provided us with a useful set of SNP and MS markers for parentage and relatedness testing. Further assessment of the utility of these markers over multiple (> three) generations and the incorporation of a larger variety of relationships among individuals (e.g. half-siblings or cousins) is strongly suggested.

## Introduction

Due to intensive protection and conservation efforts, the Southern white rhinoceros (*Ceratotherium simum simum*) have increased from a population of less than 100 at the end of the 19th century, to an estimated population of over 20,000 (Emslie, 2012). However, the illegal trade in rhinoceros horn in many parts of the world especially in Asia where the rhinoceros horns are used traditionally as material in sculptures or as drug products for medicinal purposes (Hsieh *et al*., 2003) is adding constant pressure on remaining populations. Currently, the remaining white rhino populations are being intensively managed as small isolated groups thus monitoring and maintaining genetic diversity is a key concern for long term survival of this species (Emslie and Brooks, 1999). Potential consequences of a reduction in genetic variability include (1) the inability of the species to adapt to changes in their environment and (2) inbreeding, whereby the expression of rare deleterious alleles may contribute to developmental, reproductive and immunological impairments (Pertoldi *et al*., 2007; Väli *et al*., 2008). In order to maintain genetic diversity and the species' evolutionary potential, a recovery strategy can be employed whereby gene flow amongst populations is enhanced (Lowe and Allendorf, 2010). Translocation can be considered as an option in in the case of the white rhino. However, an analysis of genetic structure is required in order to ensure that outbreeding depression due to the introduction of mal-adapted genes does not occur (Pertoldi *et al*., 2007; Väli *et al*., 2008).

Single nucleotide polymorphisms (SNPs) represent the most abundant type of DNA variation in the vertebrate genome and are distributed across the entire genome providing broader genome coverage as compared to mitochondrial DNA or microsatellites (MS) (Ryynänen and Primmer, 2006; Pertoldi *et al*., 2007). In addition, SNPs offer higher recovery of information from degraded DNA samples since the DNA target sequence in SNP-based genotyping is appreciably shorter (50-70 bp) than that in microsatellite-based genotyping (80-300 bp) (Morin *et al*., 2004; Ryynänen and Primmer, 2006; Butler *et al*., 2007; Pertoldi *et al*., 2007). In contrast to microsatellites, SNP genotyping reveals polymorphisms directly on the DNA sequence, and thus data is automatically standardized across chemistries, hardware platforms and laboratories (Smith *et al*., 2005; Glover *et al*., 2010). Furthermore, the development of high through-put genotyping platforms permits simultaneous genotyping of thousands of loci, enabling the identifications of highly diagnostic panels (Glover *et al*., 2010).

In this study, we compare the power of parentage assignment of 33 SNPs and 9 MS markers in isolation and in combination in a captive population of white rhinoceros. Development of a marker set that accurately determines parentage will provide information on the relationships and relatedness among individuals, contribute to the management of captive white rhinoceros worldwide, and additionally provide insight into mating systems in wild populations.

## Materials and Methods

Blood samples were collected from 32 white rhinoceros in South Africa. Blood aliquots were first treated by mixing 100 μL of blood with 1000 μL nuclease free water followed by centrifugation at 1500 x g for 2 min to reduce the number of red blood cells and improve DNA yields. Genomic DNA was extracted from the resulting pellet using the ZR Genomic DNA™-Tissue Mini-Prep kit (Zymo Research) following the manufacturer's instructions. A SNP enriched library was constructed using DNA from 5 individuals and digestion with Endonuclease V as previously described (Labuschagne *et al*., 2015). This protocol was used without any changes. Subsequent SNP enriched amplicons were cloned into pJET vector using the CloneJET PCR Cloning Kit (Thermo Scientific) and Z-Competent JM109 *E. coli* cells (Zymo Research). Clones containing fragments ranging from 200-700 bp were selected and sequenced utilising a Big Dye V3.1 Terminator Kit and an ABI 3500XL genetic analyser. The potential SNP loci were amplified in the 5 isolates used for the initial DNA pool. Amplification reactions were done in a final volume of 25 μL containing 30 ng DNA, 25 pM of each primer and 2X DreamTaq^®^ Green Master Mix (Thermo Scientific). Thermal cycling consisted of initial denaturation at 95 °C for 5 min, 45 cycles of denaturation at 95°C for 30 s, annealing at 55-59 °C for 30 s, extension at 72 °C for 90 s, followed by final extension at 72 °C for 10 min. Resulting amplicons were inspected on 1% agarose gels followed by purification and sequencing as described above. Sequences were inspected and aligned in CLC Bio Genomics work bench 8.0.1 (CLC bio, Denmark). Twelve resulting SNP markers were further typed in the remaining 27 isolates. GENEPOP version 4.0.10 (Raymond and Rousset, 1995) was used to test for deviations from expected Hardy-Weinberg (HW) proportions, to evaluate loci for gametic disequilibrium and to determine allelic richness. Differences in mean observed heterozygosity (Ho), mean expected heterozygosity (He) and mean number of alleles was determined using Cervus v3.03 (Kalinowski *et al*., 2007). All 32 samples were further typed for 21 previously described SNP markers through Sanger sequencing (Labuschagne *et al*., 2013, 2015).

Nine microsatellite loci: BR6 (Cunningham *et al*., 1999), DB44, DB66, DB49, DB1 (Brown and Houlden, 1999), RHI7C, RHI32A, RHI7B (Florescu *et al*., 2003), SW35 (Rohrer *et al*., 1994) were also used. Markers were selected based on previously reported polymorphism in white rhinoceros. The PCR optimization for each locus was as follows: 2 ng of template DNA, 1.5–2.5 mM MgCl_2_, 2 mM dNTP's, 1 μM forward and 1 μM reverse primer, 0.10 U*Taq*DNA polymerase and ddH_2_O to a final volume of 15 μL. PCR cycles were as follows: initial denaturing stage at 94 ºC for 3 min, followed by 30 cycles of 94 ºC for 30 s, annealing at 50-55 °C for 30 s, extension at 72 ºC for 30 s and a final step of 72 ºC for 20 min. Products were electrophoresed on an ABI Prism 3130 DNA sequencer (Applied Biosystems). Allele sizes were estimated by comparison with a Genescan™500 LIZ™ internal size standard (ABI, Foster City, CA) using the ABI programs GENESCAN (version 1.2.2.1) and GENOTYPER (version 1.1). Sanger sequencing was performed in both directions and SNP calls were only made on bases with quality scores higher than Q > 20. The SNPs all fall within the central region of the fragments where sequencing quality is the highest. In order to ensure accurate genotyping, the samples were repeated if they were homozygous, aberrant stutter patterns or spurious peaks were observed or if the profiles were below the quality score. Differences in mean observed heterozygosity (Ho), mean expected heterozygosity (He) and mean number of alleles was determined as mentioned above.

Parentage assignment was evaluated using the MS and SNP data sets individually and as a combined dataset. The software program Cervus v3.03 (Kalinowski *et al*., 2007) was implemented for parentage assignment using likelihood. The program uses multilocus parental exclusion probabilities (Selvin, 1980) and pair-wise likelihood to assign parent pairs to offspring. Cervus calculates the log-likelihood of each candidate parent being the true parent relative to an arbitrary individual and then calculates the difference between the two most likely parents (Delta, Δ). Critical values of Δ are determined by computer simulation. Using the real data for allele frequencies, simulation parameters were set at 10,000 offspring, with 100% of candidate parents sampled and a total proportion of loci typed over all individuals of 0.99, mistyping error rates = 0.01 and likelihood calculation error rates = 0.01, permitting two unscored loci. Strict confidence was set to 95% while the relaxed confidence level was 80%.

## Results

Twelve SNPs (GenBank accession numbers 1416044499-1416044509) were identified in this study across 11 loci (WR1-WR11). The primer sequences and allele frequencies of the 12 SNPs developed here together with a further 21 SNPs for the 32 individuals are listed in Table 1. The PIC ranged from 0.060 to 0.396 with a mean of 0.2742. The observed and expected heterozygosity ranged from 0.065 to 0.656 and from 0.063 to 0.520, respectively. Marked BGN deviated from Hardy-Weinberg equilibrium. Large differences between the observed and expected heterozygosity were also observed for four markers namely: WR1, WR8-Y, WR11 and Tru-3. The observed deviations may be attributed to small sample size. Linkage disequilibrium was observed between markers ACTC-2/ACTC-3, GLUT2F-1/GLUT2F-2 and Tru2-1/Tru2-2/Tru2-4/Tru2-5. Such linkage is not unexpected since these SNPs are in close proximity on the same locus. The nine MS markers, primer sequences and allele frequencies for the 32 individuals are listed in Table 2. The PIC ranged from 0.259 to 0.578 with a mean of 0.4282, while observed and expected heterozygosity ranged from 0.273 to 0.654 and from 0.298 to 0.655, respectively. None of the MS loci deviated significantly from Hardy-Weinberg equilibrium and no linkage disequilibrium was observed. Only two alleles were observed in four of the markers, while four markers exhibited three alleles and one marker, five alleles, resulting in a mean allele number (Na) of 2.7.

**Table 1:** Summary statistics for 33 SNPs in White Rhino (*Ceratotherium simum*). PIC Mean polymorphic information content, F forward primer, R reverse primer, bp base pairs, Ho observed heterozygosity, He expected heterozygosity, *F(Null)* the *F* score for the null hypothesis that the locus is in Hardy-Weinberg Equilibrium.

Locus	SNP name	Sequence length (bp)	Primer sequences (5′-3′)	PIC	Heterozygosity		*F(Null)*
					Ho	He	
WR1^a^	WR1-Y	136	F-GCAACTGAGGAGCAATCA	0.354	0.656	0.468	−0.175
			R-AGAAGCAAACTCATAAGATA				
WR2^a^	WR2-S	173	F-GTATTATGCTGAGTGATACAG	0.110	0.125	0.119	−0.023
			R-CAGGTGTAGATGCTGGA				
WR3^a^	WR3-W	562	F-CACTCACTCACCTGAGGCAC	0.314	0.406	0.396	−0.020
			R-CTGTGGAGTATATAGTCCTAGC				
WR4^a^	WR4-M	358	F-CCTGAGTAATATGACAGCAGTCC	0.330	0.531	0.424	−0.119
			R-GTAAGGCCTGCTGCTCTTAG				
WR5^a^	WR5-K	349	F-CTTCTCCTGTTACTGCATGGTCAC	0.176	0.219	0.198	−0.052
			R-GTCAGTGGTGCCAATATGCAAG				
WR6^a^	WR6-Y	586	F-GACTCGCCCTTTGTGAAAGTG	0.134	0.156	0.146	−0.032
			R-CTGCATTGTTGCCTGGTTC				
WR7^a^	WR7-R	406	F-GAGCTGCTGCTCAGCAGAG	0.314	0.469	0.396	−0.091
			R-GTACCTCTGAGAAGCCACTAG				
WR8^a^	WR8-Y	485	F-GTGCTTCTTCACAGCTGTAG	0.244	0.344	0.289	−0.091
	WR8-R		R-GATACGTGTGTTTGGAGTGG	0.134	0.156	0.146	−0.032
WR9^a^	WR9-K	197	F-GACTTCCAAATGTAAGAAGGTG	0.314	0.344	0.396	0.063
			R-CAAGTTTCTTTGCTGAATGTTTGC				
WR10^a^	WR10-M	333	F-CACTGTATACCAAACAAAATGG	0.349	0.500	0.458	−0.051
			R-CTCACAATTCTGCAATCTGG				
WR11^a^	WR11-W	296	F-GGGTCACCTTAGGTAGG	0.359	0.250	0.476	0.304
			R-GAGGAATAACACAAGTAACAACG				
MGF^b^	MGF-1	820	F-ATCCATTGATGCCTTCAAGG	0.362	0.516	0.482	−0.042
	MGF-2		R-CTGTCATTCCTAAGGGAGCTG	0.060	0.065	0.063	−0.007
ACTC^b^	ACTC-1	875	F-GCCCTGGATTTTGAGAATGAGAT	0.353	0.516	0.465	−0.059
	ACTC-2			0.310	0.452	0.389	−0.082
			R-ACGATCAGCAATACCAGGGTACA				
	ACTC-3			0.358	0.484	0.474	−0.018
BGN^b^	BGN	647	F-CTCCAAGAACCACCTGGTG	0.363	0.156	0.484	0.505
			R-TTCAAAGCCACTGTTCTCCAG				
GLUT2^b^	GLUT2F-1	301	F-TGGATGAGTTATGTGAGCAT	0.369	0.594	0.496	−0.098
	GLUT2F-2		R-GACTTTCCTTTGGTTTCTGG	0.369	0.594	0.496	−0.098
KIT^b^	KIT-1	641	F-CCTGTGAAGTGGATGGCACC	0.176	0.156	0.198	0.109
	KIT-2		R-GCATCCCAGCAAGTCTTCAT	0.155	0.188	0.173	−0.042
Hpa-1^c^	Hpa-1-K	605	F- GGGATCATTCATTCATTCAGCTG	0.310	0.258	0.389	0.194
			R- GGAACTCCAGAAGCCACG				
Tru-1^c^	Tru-1-K	380	F- GAGAGCTTTCTCTCCTGAT	0.085	0.094	0.091	−0.014
			R- GAACTGGAAGTGTGTCAAC				
Tru-2^c^	Tru-2-1-S	345	F- CCAGCATGGCTAGCATGC	0.396	0.531	0.520	−0.017
	Tru-2-2-R			0.375	0.531	0.507	−0.030
	Tru-2-3-Y			0.134	0.156	0.146	−0.032
	Tru-2-4-Y		R- CAGCCCTATCCGTGACTTTC	0.375	0.531	0.507	−0.030
	Tru-2-5-R			0.375	0.531	0.507	−0.030
	Tru-2-6-M			0.134	0.156	0.146	−0.032
Tru-3^c^	Tru-3-M	335	F- GGCTCTGTTTGCTTGTCTG	0.294	0.281	0.365	0.121
			R- CTTAGTGCTAGATTCTGCATG				
Tru-4^c^	Tru-4-K	362	F- GTAGAACCTTCATCTCTGC	0.258	0.375	0.310	−0.101
			R- GCAGCTGCATTATATCCAC				
Tru-5^c^	Tru-5-W	193	F- CTTGTGCTATTCTTCACTGTC	0.327	0.452	0.419	−0.045
			R- CAAGACGTCCACTGCAC				

a) This study;

b) Labuschagne *et al*. (2013);

c) Labuschagne *et al*. (2015).

**Table 2:** List of nine microsatellite loci used for DNA profiling in white rhino.

Locus	Primer sequences (5′-3′)	Allele size range	Allele no.	PIC	Heterozygosity		*F(Null)*	Reference
					Ho	He		
SW35	F-TCAAGTTGGAGAGTCTGAGGC	127-133	2	0.417	0.545	0.535	−0.0310	Rohrer*et al*., 1994
	R-AAGACTGCCCACCAAATGAG							
BR6	F-TCATTTCTTTGTTCCCCATAGCAC	133-153	3	0.474	0.424	0.529	+0.0894	Cunningham*et al*., 1999
	R-AGCAATATCCACGATATGTGAAGG							
DB44	F-GGTGGAATGTCAAGTAGCGG	173-181	2	0.363	0.469	0.441	−0.0525	Brown and Houlden, 1999
	R-CTTGTTGCCCCATCCCTG							
DB66	F-CCAGGTGAAGGGTCTTATTATTAGC	201-203	3	0.416	0.452	0.531	+0.0595	Brown and Houlden, 1999
	R-GGATTGGCATGGATGTTACC							
RHI7C	F-TGAACTCTGATGGAAATGAG	247-255	3	0.480	0.500	0.555	+0.0145	Florescu*et al*., 2003
	R-AAACAGGTCTTGATTAGTGC							
DB49	F-GTCAGGCATTGGCAGGAAG	159-163	3	0.578	0.654	0.655	−0.0266	Brown and Houlden, 1999
	R-CAGGGTAAGTGGGGGTGC							
RHI32A	F-CAGTCCTGCTGCATAAATCTC	234-248	2	0.406	0.548	0.513	−0.0559	Florescu*et al*., 2003
	GCAGTACAGCTAGAATCACC							
RHI7B	F-CCTCTGTGATTAAGCAAGGC	261-269	5	0.461	0.438	0.519	+0.0799	Florescu*et al*., 2003
	R-ATGAACAGGAAGGAAGACGC							
DB1	F-AGATAATAATAGGACCCTGCTCCC	129-131	2	0.259	0.273	0.298	+0.0120	Brown and Houlden, 1999
	R-GGAGGTTTATTGTGAATGAGGC							

**Figure 1:**
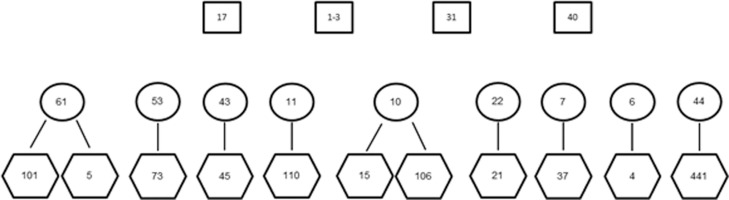
Diagram illustrating the known relationships between 24 rhino samples. Rectangles indicate potential paternal candidates, ovals the maternal samples and hexagons the offspring.

The 32 individuals consisted of 11 known mother/offspring groups with two mothers having two offspring as illustrated in Figure 1. There was a further seven juvenile samples, which did not have known mothers in the data set as well as one adult female without any offspring. The data set included four adult male samples presumed from observational data to be the possible paternal candidates for all 11 juvenile samples with known mothers. Parentage analysis was conducted with all ten adult females as maternal candidates group, all four adult males as paternal candidate group against all 18 juveniles as offspring set. The summary of parentage assignment for maternal candidates is given in Table 3 and paternal candidates in Table 4. The SNP dataset achieved a combined first parent non-exclusion probability of 0.0889, the MS data set 0.2755 and the combined data sets 0.0153. The combined second parent non-exclusion probabilities were 0.0072, 0.0735 and 0.0001 for SNP, MS and combined data sets respectively. Using the SNP data set, all 11 juveniles were correctly assigned to their mothers with no pair loci mismatching noted. The MS data set correctly assigned ten out of the 11 parent offspring pairs. No pair loci mismatching was noted in any MS assignments including the wrong assignment of maternal candidate WR-22 to juvenile WR-110. In order to assess the effect of missing MS loci on the assignment of parentage, analysis on the assignment of mothers to a subset of samples; WR101 and WR44.1 was conducted. In both cases a reduction of MS loci from nine to five maintained correct assignment (positive LOD scores), however an absence of three markers resulted in a drop of the pair confidence from 95% to 80%. All 11 juveniles were correctly assigned using the combined data set with ten assignments having confidence of 95% and one with 80%.

**Table 3:** Cervus parentage assignment for maternal candidates showing the two most likely candidates. *=95% confidence; +=80% confidence; incorrect assignments marked in grey.

	SNP				MS				Combined			
Offspring ID	Candidate mother ID	Pair loci compared	Pair loci mismatching	Pair LOD score	Candidate mother ID	Pair loci compared	Pair loci mismatching	Pair LOD score	Candidate mother ID	Pair loci compared	Pair loci mismatching	Pair LOD score
WR-5	WR-61	33	0	6.97 (*)	WR-61	8	0	0.69	WR-61	41	0	7.66 (*)
	WR-61	33	0	6.97 (*)	WR-61	8	0	0.69	WR-61	41	0	7.66 (*)
WR-101	WR-61	33	0	4.29 (*)	WR-61	9	0	5.76 (*)	WR-61	42	0	10.05 (*)
	WR-22	33	1	−2.12	WR-61	9	0	5.76 (*)	WR-22	42	2	−5.21
WR-73	WR-53	33	0	5.08 (*)	WR-53	7	0	0.32	WR-53	40	0	5.43 (*)
	WR-53	33	0	5.08 (*)	WR-43	7	0	1.90	WR-53	40	0	5.43 (*)
WR-45	WR-43	33	0	6.34 (*)	WR-43	8	0	1.25 (+)	WR-43	41	0	7.62 (*)
	WR-53	33	0	3.92	WR-11	8	0	−0.58	WR-53	41	1	0.21
WR-110	WR-11	33	0	1.81 (*)	WR-22	6	0	0.94	WR-11	39	0	0.06 (+)
	WR-53	33	1	−1.80	WR-22	6	0	0.94	WR-53	39	1	−2.82
WR-15	WR-7	33	3	−9.02	WR-10	8	0	0.77	WR-10	41	0	4.73 (*)
	WR-10	33	0	3.97 (*)	WR-10	8	0	0.77	WR-10	41	0	4.73 (*)
WR-106	WR-10	33	0	3.42 (*)	WR-10	8	0	1.98 (+)	WR-10	41	0	5.40 (*)
	WR-10	33	0	3.42 (*)	WR-61	9	1	−1.07	WR-10	41	0	5.40 (*)
WR-21	WR-44	33	0	4.61	WR-22	7	0	1.31	WR-22	40	0	7.16 (*)
	WR-22	33	0	5.84 (*)	WR-22	7	0	1.31	WR-22	40	0	7.16 (*)
WR-37	WR-7	33	0	4.72 (*)	WR-7	7	0	2.06	WR-7	40	0	6.80 (*)
	WR-7	33	0	4.72 (*)	WR-7	7	0	2.06	WR-7	40	0	6.80 (*)
WR-4	WR-6	33	0	4.79 (*)	WR-6	7	0	1.10	WR-6	40	0	4.91(*)
	WR-11	33	1	−1.48	WR-6	7	0	1.10	WR-43	40	3	−10.37
WR-44.1	WR-44	33	0	5.70 (*)	WR-44	9	0	3.94 (*)	WR-44	42	0	9.00 (*)
	WR-53	33	0	3.56	WR-57	9	0	0.57	WR-44	42	0	9.00 (*)

**Table 4:** CERVUS parentage assignment for paternal candidates showing the two most likely candidates. *=95% confidence; +=80% confidence

	SNP				MS				Combined			
Offspring ID	Candidate father ID	Pair loci compared	Pair loci mismatching	Pair LOD score	Candidate father ID	Pair loci compared	Pair loci mismatching	Pair LOD score	Candidate father ID	Pair loci compared	Pair loci mismatching	Pair LOD score
WR-5	WR-17	33	1	−0.38	WR-17	8	0	0.32	WR-17	41	1	−0.06
	WR-1-3	33	1	−3.93	WR-1-3	8	0	0.95 (+)	WR-1-3	41	1	−2.98
WR-101	WR-31	32	0	4.89 (*)	WR-31	9	0	−0.67	WR-31	41	0	4.22 (*)
	WR-31	32	0	4.89 (*)	WR-40	9	1	−5.88	WR-31	41	0	4.22 (*)
WR-73	WR-17	33	1	−1.88	WR-17	7	0	1.08 (+)	WR-17	40	1	−0.80
	WR-31	32	1	−0.99	WR-31	7	0	0.86	WR-31	39	1	−0.11
WR-45	WR-17	33	0	1.86 (*)	WR-17	8	0	1.70 (*)	WR-17	41	0	3.57 (*)
	WR-17	33	0	1.86 (*)	WR-17	8	0	1.70 (*)	WR-17	41	0	3.57 (*)
WR-110	WR-31	32	0	6.01 (*)	WR-1-3	6	0	−0.02	WR-31	38	0	5.43(*)
	WR-31	32	0	6.01 (*)	WR-17	9	0	−0.64	WR-31	38	0	5.43(*)
WR-15	WR-17	33	3	−9.91	WR-17	9	0	0.07 (+)	WR-17	42	3	−9.84
	WR-31	32	3	−15.33	WR-1-3	9	0	−1.43	WR-1-3	42	2	−9.02
WR-106	WR-17	33	1	0.90 (*)	WR-40	9	0	0.73 (+)	WR-31	41	3	−13.40
	WR-31	32	1	−5.71	WR-40	9	0	0.73 (+)	WR-17	42	4	−10.45
WR-21	WR-17	33	0	3.63 (*)	WR-31	7	0	1.52 (+)	WR-17	40	1	−0.45
	WR-17	33	0	3.63 (*)	WR-40	7	0	0.93	WR-31	39	2	−6.23
WR-37	WR-31	32	0	1.63	WR-31	9	0	0.21 (+)	WR-31	39	0	1.86 (*)
	WR-1-3	33	0	2.63 (*)	WR-40	7	0	−0.37	WR-1-3	40	1	−1.82
WR-4	WR-31	32	1	0.10	WR-31	7	0	1.17	WR-31	39	1	0.34(*)
	WR-31	32	1	0.10	WR-40	7	0	0.48	WR-31	39	1	0.34(*)
WR-44.1	WR-17	33	1	−1.73	WR-1-3	9	0	2.36 (*)	WR-17	42	1	−2.73
	WR-17	33	1	−1.73	WR-1-3	9	0	2.36 (*)	WR-1-3	42	4	−12.38

Using the SNP data set, six paternal allocations could be made with 95% confidence. Using the MS data set, two paternal allocations can be made with 95% confidence and six with 80% confidence. Two of the allocations with positive scores correspond between the two data sets. Paternal allocations on the combined dataset were conducted in two ways; where maternal assignments were fixed and without known mothers. Using the combined data set with unknown mothers, five paternal allocations could be made with 95% confidence (Table 4). In the case of fixed maternal assignment, six paternal allocations could be determined with 95% confidence. In this analysis, all allocations determined with unknown mothers remained, however an additional assignment could be made with 95% confidence (WR-17 assigned as the father of WR-5, pair LOD score 2.63). Table 5 includes the parentage assignments when siblings WR-101/WR-5 and WR-15/WR-106 are included in the pool of maternal candidates. Using only SNP data, the correct maternal candidates are assigned to WR-5 and WR-106. WR-5 was wrongly assigned as best maternal candidate for both WR-101 and WR-15. Using only MS data the assignments are correct except for WR-101 which has a higher LOD score than the true mother for WR-106. Using the combined data sets the assignments are all correct with 95% confidence. All other assignments remained as stated in Table 3.

**Table 5:** Cervus parentage assignment for maternal candidates including siblings showing the two most likely candidates. *=95% confidence; +=80% confidence; incorrect assignments marked in grey.

	SNP				MS				Combined			
Offspring ID	Candidate mother ID	Pair loci compared	Pair loci mismatching	Pair LOD score	Candidate mother ID	Pair loci compared	Pair loci mismatching	Pair LOD score	Candidate mother ID	Pair loci compared	Pair loci mismatching	Pair LOD score
WR-5	WR-61	33	0	6.97 (*)	WR-61	8	0	0.69	WR-61	41	0	7.66 (*)
	WR-61	33	0	6.97 (*)	WR-61	8	0	0.69	WR-61	41	0	7.66 (*)
WR-101	WR-5	33	0	4.76	WR-61	9	0	5.76 (*)	WR-61	42	0	10.05 (*)
	WR-61	33	0	4.29	WR-61	9	0	5.76 (*)	WR-5	41	2	−2.90
WR-15	WR-7	33	3	−9.02	WR-10	8	0	0.77	WR-10	41	0	4.73 (*)
	WR-5	33	1	1.18	WR-10	8	0	0.77	WR-10	41	0	4.73 (*)
WR-106	WR-10	33	0	3.42 (*)	WR-101	9	0	2.73 (+)	WR-10	41	0	5.40 (*)
	WR-10	33	0	3.42 (*)	WR-10	8	0	1.98	WR-10	41	0	5.40 (*)

## Discussion

Together with the 12 new SNPs identified in this study, 33 SNPs are now available for white rhino (Labuschagne *et al*., 2013, 2015). The SNPs, were discovered through random selection and sequencing of cloned fragments from a SNP enriched library. Ascertainment bias is often a concern when using SNPs in population studies, with bias introduced by heterogeneity in the SNP discovery process, varying sample sizes or differences in sample composition leading to underestimation or overestimation of the frequency of SNPs (Nielsen and Signorovitch, 2003; Clark *et al*., 2005). Ascertainment of SNPs through discovery in particular populations or genomic regions does not bias the results of parentage inference in any way since the parentage analysis is not concerned with the inference of evolutionary history (Anderson and Garza, 2006). In effect, SNP ascertainment leads to an advantage in parentage inference, since ascertainment typically leads to an overrepresentation of intermediate allele frequency SNPs, the type of loci that are most powerful for parentage (Anderson and Garza, 2006). The SNP loci presented here contain extra flanking data to allow for Sanger sequencing. Shorter amplicons may be designed in the future to optimise their utility in degraded DNA samples.

To our knowledge this is the first study to employ SNP and MS markers for parentage analysis in white rhino. The current SNP set outperform the MS set during maternal assignment, where all assignments were correct while the MS data set allocated one maternal sample incorrectly. In general, assignments made with the SNP data set had higher confidence than those with the MS set. Confidence levels increased when combining the two data sets. The increased accuracy of the SNP markers in this study over MS markers can be attributed to the greater marker numbers in the SNP data set and low allele numbers of the MS markers. It is apparent that low levels of genetic diversity characterise white rhino populations and the results from our study (Na=2.7; PIC=0.4282) are consistent with other studies making use of MS markers. Harper *et al*. (2013) reported Na=2.722 and PIC=0.329 for 367 rhino samples, while Guerier *et al*. (2012) reported Na=2.72 and PIC=0.357 in a sample set of 31 individuals. Florescu *et al*. (2003) observed higher values, Na=2.8 and PIC=0.4812 in a sample set of 30 individuals, but selected specifically for highly polymorphic loci, which may account for the elevated genetic diversity estimates in their data. The low levels of genetic diversity observed in white rhinos may be attributed to the small (20-40 individuals) founder population and subsequent bottleneck (Walker and Walker, 2012),

Challenges to parentage assessment can arise when family members other than the parents of the offspring are included in the pool of candidate parents (Jones and Ardren, 2003). Inclusion of either half- or full-siblings in the pool of candidate parents may pose the most problematic situations (Jones and Ardren, 2003). In the current study two pairs of siblings were available to evaluate the effect on assignment when included in the parental pool. Inclusion led to some wrong assignments when using the two marker sets separately, but not in the combined data set. It would seem that the combined data set has enough discriminating power for accurate assignment in the current population even when siblings are included in the parental pool, however as relatedness levels increase so should the number of markers. In extremely inbred populations this may reach prohibitive numbers. Further assessment of the utility of these markers over multiple (> three) generations and the incorporation of a larger variety of relationships among individuals (e.g. half-siblings or cousins) as well as a larger set of samples is strongly suggested.
